# Morphology, Classification, and Distribution of the Projection Neurons in the Dorsal Lateral Geniculate Nucleus of the Rat

**DOI:** 10.1371/journal.pone.0049161

**Published:** 2012-11-05

**Authors:** Changying Ling, Michael L. Hendrickson, Ronald E. Kalil

**Affiliations:** 1 Department of Surgery, School of Medicine and Public Health, University of Wisconsin-Madison, Madison, Wisconsin, United States of America; 2 W.M. Keck Laboratory for Biological Imaging, School of Medicine and Public Health, University of Wisconsin-Madison, Madison, Wisconsin, United States of America; 3 Department of Ophthalmology and Visual Sciences, School of Medicine and Public Health, University of Wisconsin-Madison, Madison, Wisconsin, United States of America; University of Edinburgh, United Kingdom

## Abstract

The morphology of confirmed projection neurons in the dorsal lateral geniculate nucleus (dLGN) of the rat was examined by filling these cells retrogradely with biotinylated dextran amine (BDA) injected into the visual cortex. BDA-labeled projection neurons varied widely in the shape and size of their cell somas, with mean cross-sectional areas ranging from 60–340 µm^2^. Labeled projection neurons supported 7–55 dendrites that spanned up to 300 µm in length and formed dendritic arbors with cross-sectional areas of up to 7.0×10^4^ µm^2^. Primary dendrites emerged from cell somas in three broad patterns. In some dLGN projection neurons, primary dendrites arise from the cell soma at two poles spaced approximately 180° apart. In other projection neurons, dendrites emerge principally from one side of the cell soma, while in a third group of projection neurons primary dendrites emerge from the entire perimeter of the cell soma. Based on these three distinct patterns in the distribution of primary dendrites from cell somas, we have grouped dLGN projection neurons into three classes: bipolar cells, basket cells and radial cells, respectively. The appendages seen on dendrites also can be grouped into three classes according to differences in their structure. Short “tufted” appendages arise mainly from the distal branches of dendrites; “spine-like” appendages, fine stalks with ovoid heads, typically are seen along the middle segments of dendrites; and “grape-like” appendages, short stalks that terminate in a cluster of ovoid bulbs, appear most often along the proximal segments of secondary dendrites of neurons with medium or large cell somas. While morphologically diverse dLGN projection neurons are intermingled uniformly throughout the nucleus, the caudal pole of the dLGN contains more small projection neurons of all classes than the rostral pole.

## Introduction

For many years, the retino-geniculo-cortical pathway in mammals has been the site of studies examining neuronal development, plasticity, and regeneration, and often these studies have involved the dorsal lateral geniculate nucleus (dLGN). The organization of the dLGN and the structure and function of dLGN neurons have been described in many species [1], [2]. In particular, dLGN neurons in the rat have been studied extensively in normal adults and during development [3] and aging [4] using a large variety of techniques. These studies have helped to clarify the structure [3]–[11] and function [12]–[18] of dLGN cells, their afferent [19]–[26] and efferent [27]–[32] connections, the hidden laminar organization [33], and the topological representation of the visual field in the dLGN [34]. Despite this wealth of information, very little attention has been paid to describing the morphology of confirmed dLGN projection neurons in the rat. Indeed, the structure of a confirmed dLGN projection neuron appears to have been described previously in only one paper [10].

dLGN projection neurons in adult rats have been used as an *in vivo* model to study the neuronal responses to axotomy, and two studies have demonstrated that approximately 70% of the dLGN neurons in adult rats die within a week after removal of the visual cortex [35], [36]. However, the morphological changes that axotomized projection neurons undergo before dying are not well understood, nor is it clear whether all dLGN projection neurons react similarly to axotomy. To answer these questions, it is necessary first to understand the detailed morphology of normal dLGN projection neurons, before carefully characterizing the effects of axotomy on their structure. We have recently completed this analysis by first refining a biotinylated dextran amine (BDA) tracing technique in order to label dLGN projection neurons retrogradely in fine detail [37]. Here we present a description of the detailed morphology of identified dLGN projection neurons in the normal adult rat, and propose a classification of these neurons into three distinct morphological classes based on differences in the spatial structure of their dendritic arbors. These results then were used as the basis for comparing the dendritic architecture of normal projection neurons with that of axotomized projection neurons [38].

## Materials and Methods

### Ethics Statement

#### Research Animals

All animal handling and procedures were performed in accordance with protocols for these studies that have been approved by the Institutional Animal Care and Use Committee at the University of Wisconsin-Madison. All surgery was performed aseptically under deep anesthesia, and every attempt was made to minimize pain and discomfort.

### Research Animals

#### Labeling of dLGN Projection Neurons with BDA

**Figure 1:**
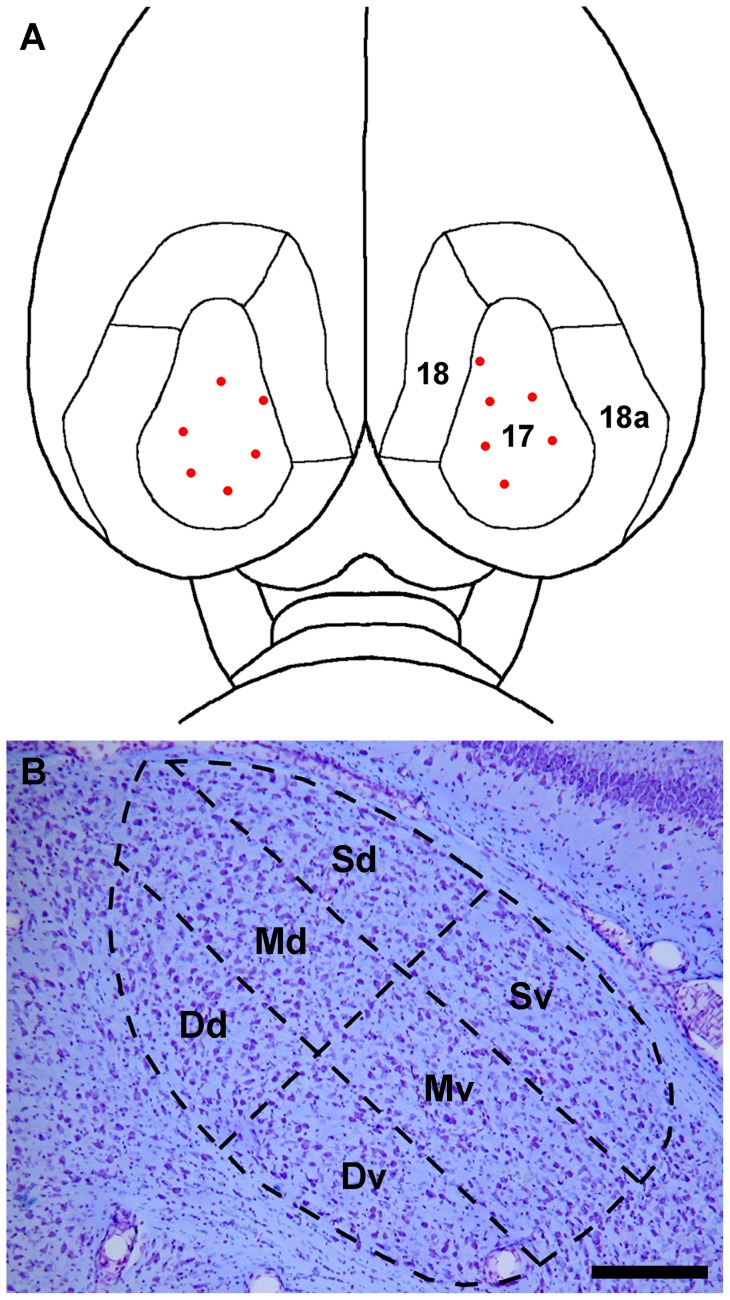
Dorsal view of the rat brain showing visual cortical areas 17, 18, and 18a. (**A**) The sites of biotinylated dextran amine (BDA) injections are represented by red dots. (**B**) A schematic drawing representing a coronal section of the dLGN near the rostro-caudal midpoint of the nucleus, A–P plane 5, superimposed on a corresponding cresyl violet stained section of the dLGN. The dLGN is divided arbitrarily into six sectors: Sd, superficial dorsal; Sv, superficial ventral; Md, mid-dorsal; Mv, mid-ventral; Dd, deep dorsal; Dv, deep ventral. Scale bar: 3.0 mm for **A** and 250 µm for **B**.

Thirty-seven adult male Holtzman rats (250–275 grams) were used in this study. Rats were anesthetized initially with an intramuscular injection of a mixture of 70 mg/kg of ketamine HCl and 7 mg/kg of xylazine. The animals then were placed in a stereotaxic head holder, and surgical anesthesia was maintained with 1–2% isoflurane in oxygen at a flow rate of 300 mL/min. Under aseptic conditions, bone flaps were cut bilaterally to expose the dorsal surface of the visual cortex, cortical areas 17, 18, and 18a as previously defined [27], [29], [32]. A 5% w/v solution of 3,000 MW BDA (Invitrogen) in double-distilled H_2_O was pressure-injected into the visual cortex through a glass micropipette with a tip diameter of approximately 10 µm. Typically, five 0.1 µL injections of BDA were made bilaterally in the visual cortex at a depth of 0.8 mm below the surface of the brain (Figure 1A). The injections were aimed at the approximate center of cortical layer IV within cortical area 17, and each injection was made slowly over a period of 10 minutes. When each injection was completed, the pipette was left in place for an additional 2 minutes before withdrawing it to reduce back-flow of the injected solution. The bone flaps then were replaced and the scalp incision was closed with wound clips. The rats were returned to their home cages after recovery on a warming pad.

Three days after the BDA injections, the animals were anesthetized deeply with a mixture of 135 mg/kg of ketamine HCl and 14 mg/kg of xylazine and perfused through the heart with 0.9% NaCl followed by 4% paraformaldehyde in 0.1 M phosphate buffer (pH 7.4). The brains were post-fixed overnight in 4% paraformaldehyde and then sectioned coronally at 75 µm with a vibrating blade microtome (Leica, VT 1000S).

#### Cytochemistry

Retrogradely transported BDA was detected in the dLGN by using an avidin-biotin-peroxidase complex (Vector Laboratories, Elite ABC Kit), and visualized using diaminobenzidine (DAB) as a chromogen. In brief, free-floating sections were blocked in 0.1% bovine serum albumin and 0.5% Triton X-100 in 0.1 M PBS for one hour at room temperature. The sections then were washed thoroughly in PBS and incubated in a solution of avidin-biotin-peroxidase complex (1∶100 in PBS) for 2 hours at room temperature or overnight at 4°C. The sections were washed once again in PBS and then reacted with DAB (0.005% DAB in 0.1 M PBS containing 0.006% H_2_O_2_) for 5–10 minutes. These sections were washed thoroughly in PBS, mounted on slides, de-fatted in xylene, and rehydrated in preparation for gold/silver intensification. The intensification was done by immersing the sections first in 1.42% silver nitrate for one hour at 56°C, then in 0.2% gold chloride for 10 minutes at room temperature, and lastly in 5% sodium thiosulfate for 5 minutes at room temperature, with thorough washing in PBS between each step. The intensified sections were dehydrated in graded alcohol, cleared in xylene, and coverslipped.

#### Morphological and Quantitative Analysis

All sections were examined under a brightfield microscope with a 100X oil immersion objective (NA  = 1.4). Projection neurons labeled with BDA that lay at or near the surface of the section were discriminated easily by their truncated dendrites and discontinuities between apparent proximal dendrites and their respective cell somas. These labeled cells were excluded from further analysis. Projection neurons with cell somas and dendrites that appeared intact and were labeled completely with BDA were used for the detailed analysis and classification of dLGN projection neurons.

BDA-labeled projection neurons that displayed a cell soma with contiguous proximal dendrites within the plane of the 75 µm section studied were used in the analysis of the size and the distribution of projection neuron cell somas. The somas of completely labeled neurons were traced with the aid of a drawing tube and the location of each projection neuron in the dLGN was recorded by assigning it a coordinate within the dLGN. The cell soma of each classified, reconstructed projection neuron was moved until it was directly under a cross hair in the microscope's ocular. The X and Y coordinates for the cell soma's location then were read from the microscope's stage verniers and recorded. This process was completed for each projection neuron that was reconstructed in each section through the dLGN that was studied. The locations of the classified, reconstructed projection neurons with respect to the borders of the dLGN in each section were determined by assigning an X and Y coordinate to the midpoints of the lateral, medial, ventral and dorsal borders of the dLGN. The A-P plane of each section studied was determined by comparing the section with a comparable one in an atlas of the rat brain [39]. This analysis allowed the location of each projection neuron that had been classified and reconstructed to be noted with respect to the borders of the dLGN at different A–P levels.

For each completely labeled projection neuron, an outline of the dendritic arbor that lay entirely within the plane of the section studied was traced by connecting the terminal points of dendritic branches to adjacent terminal points in order to circumscribe an area that was taken as a measure of dendritic arbor size. The tracings were digitized by scanning them and then were analyzed by importing them into imaging software (MetaMorph). The tracings of cell somas also were digitized, imported and analyzed. The cross-sectional areas of dendritic arbors and cell somas were computed directly from the images created by the software. Both the number of primary dendrites and total dendrites for each fully labeled projection neuron also were counted. The number of total dendrites is equal to the sum of the primary dendrites and terminal dendritic branches.

To estimate the distribution of dLGN projection neurons of different cell soma sizes, the dLGN was divided rostrocaudally into nine equally spaced coronal planes. A–P plane 1 coincided with the anterior pole of the nucleus, which is located approximately 3.6 mm caudal to bregma, and A–P plane 9 with the posterior pole, 5.3 mm caudal to bregma [39]. At the rostrocaudal midpoint of the dLGN, A–P plane 5, the nucleus was divided into six roughly equal sectors, which are referred to as “superficial” (S), “middle” (M), or “deep”(D) relative to the dorsolateral surface of the rat dLGN, and “dorsal” (d) or “ventral” (v) relative to an imaginary line through the center of the nucleus that intersects the dorsolateral perimeter at approximately 90° (Figure 1B).

The sizes and distribution of all fully-labeled projection cell somas located in A–P planes 2 (anterior), 5 (middle), and 8 (posterior) were analyzed. In addition, all completely labeled dLGN projection neurons, regardless of their location, were examined with respect to the number and distribution of their dendrites, and the relationship of these two cytological features to cell soma size. The mean cross-sectional areas of the BDA-labeled neuronal somas in each of the six sectors of the dLGN at A–P plane 5 also were calculated.

### Statistical Analysis

All mean values are expressed ± the standard error of the mean. Differences between the dLGN sectors with respect to cell soma size, and the relation between: (a) cell soma size and dendritic arbor size, (b) cell soma size and dendrite number; and (c) dendrite number and dendritic arbor size were evaluated with unpaired Student's two tailed *t*-tests. Differences between means were considered significant when they exceeded p<0.01.

## Results

### Labeling Characteristics

#### Overview

**Figure 2:**
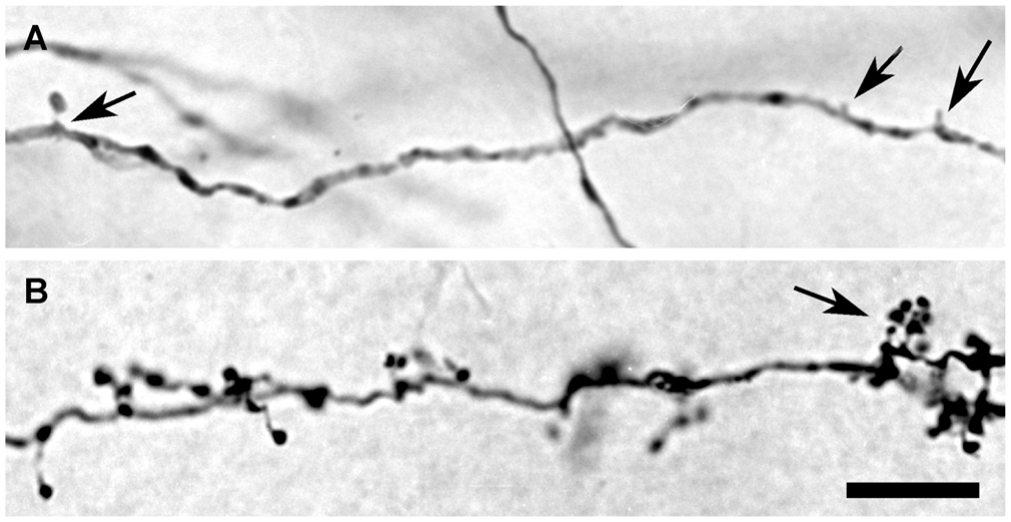
Micrographs showing the morphology of BDA-labeled dendrites and axons in the dLGN. (**A**) A dendrite of a dLGN projection neuron bears a spine-like appendage (arrow on the left) at its proximal segment and several tufted appendages (arrows on the right) at its distal segment. (**B**) A corticogeniculate axon terminates in the dLGN. The axon displays many individual terminal boutons and ends with a bouton cluster (arrow). Scale bar: 10 µm.

Three days following the injection of BDA into area 17 of the visual cortex, labeled neurons were seen in the dLGN of all animals studied. As dLGN projection neurons in the rat terminate almost exclusively in area 17 [29], it is reasonable to conclude that BDA injections in area 17 label all classes of projection neurons throughout the dLGN. Each BDA injection resulted in a labeled patch in the dLGN that consisted of both retrogradely labeled dLGN projection neurons and anterogradely labeled corticogeniculate axons. Within the patches, BDA-labeled dendrites could be discriminated from labeled axons by their morphological differences (Figure 2).

#### Morphology of dLGN Projection Neurons

Many BDA-labeled projection neurons were found in the dLGN of each animal studied. Some of the labeled neurons had solidly filled cell somas and proximal dendrites, but only lightly or granularly labeled distal dendrites. The dendritic arbors of these cells were not analyzed. Other labeled neurons, although well delineated, were difficult to visualize because of interference created by other labeled cells or axons and their terminals in the immediate vicinity. Cells that could not be isolated clearly for visualization were excluded from further consideration. When all labeled neurons not suitable for complete analysis had been excluded, 149 labeled projection neurons distributed throughout the dLGN that appeared intact and completely labeled with BDA were included in the analysis of dLGN projection neuron structure.

#### Dendrites and Axons

**Figure 3:**
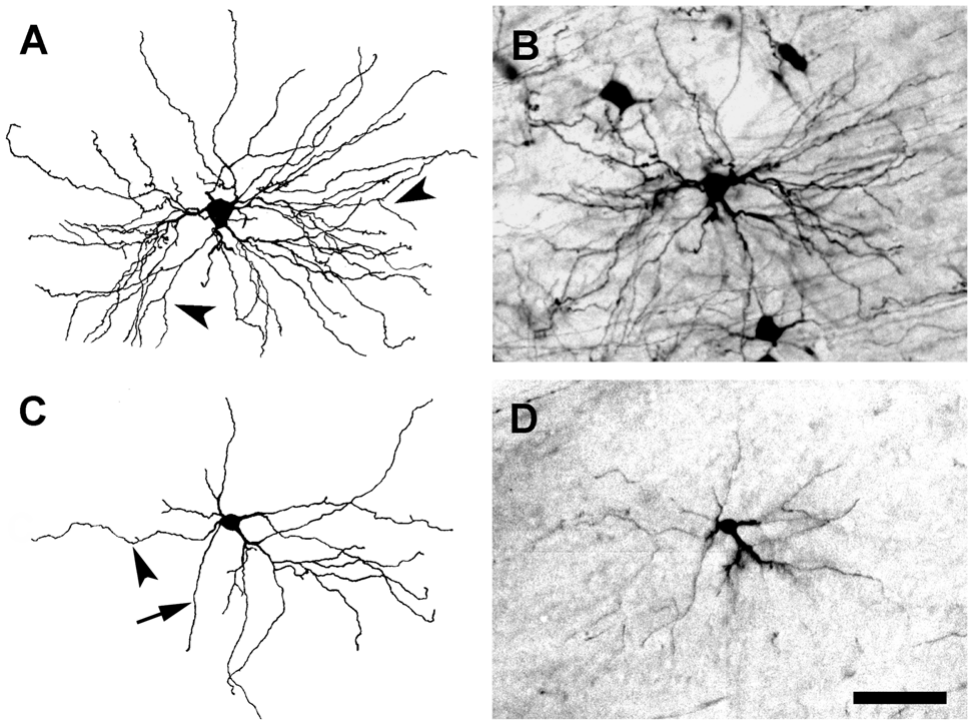
BDA-labeled radial cells. Each radial cell has more than 4 primary dendrites that array evenly around its cell soma. Large radial cells have many more dendrites (∼50) than small radial cells (∼15). (**A**) Camera lucida drawing of a large radial cell shown in **B**. Arrowheads indicate the points from which tertiary dendritic branches arise. (**B**) Micrograph of the radial cell illustrated in **A**. (**C**) Camera lucida drawing of a small radial cell. The arrowhead indicates a small caliber primary dendrite that terminates without branching. The arrow points to the neuron's axon. (**D**) Micrograph of a radial cell illustrated in **C**. Scale bar: 50 µm.

**Figure 4:**
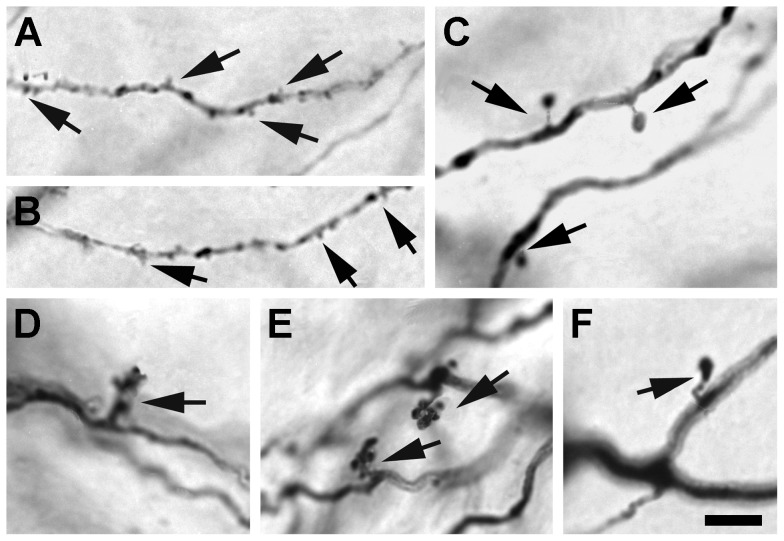
Micrographs showing three types of dendritic appendages of dLGN projection neurons. (**A** and **B**) Tufted appendages (arrows), (**C** and **F**) spiny appendages (arrows), and (**D** and **E**) grape-like appendages (arrows). Scale bar: 10 µm.

**Figure 5:**
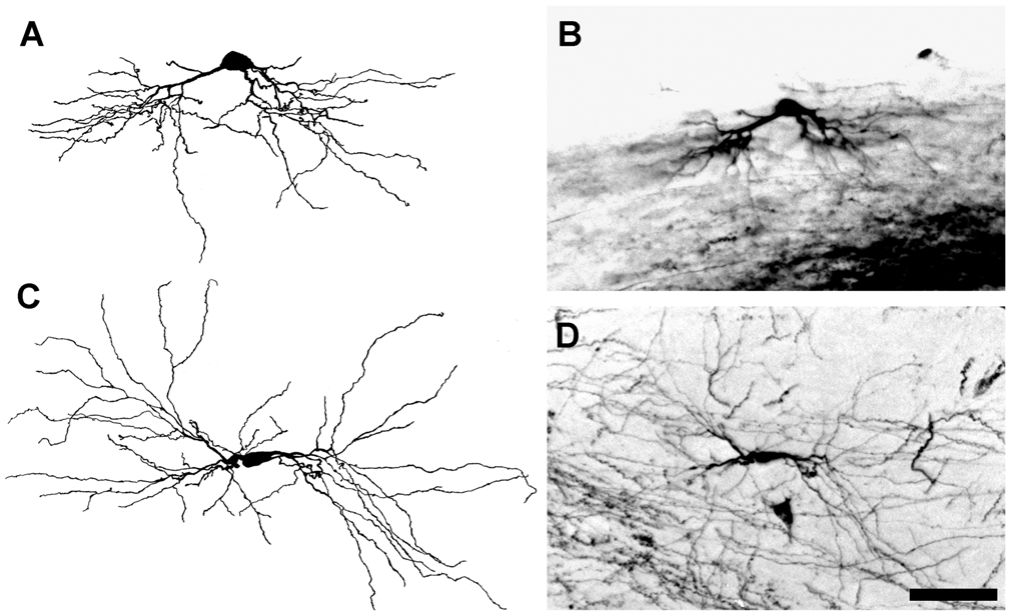
BDA-labeled bipolar cells. Each bipolar cell extends primary dendrites from two locations on opposite sides of the cell soma. (**A**) Camera lucida drawing of a bipolar neuron located in the dorsolateral dLGN. (**B**) Micrograph of the neuron in **A**. (**C**) Camera lucida drawing of a bipolar neuron in the ventromedial dLGN. (**D**) Micrograph of the neuron shown in **C**. Scale bar: 50 µm.

**Figure 6:**
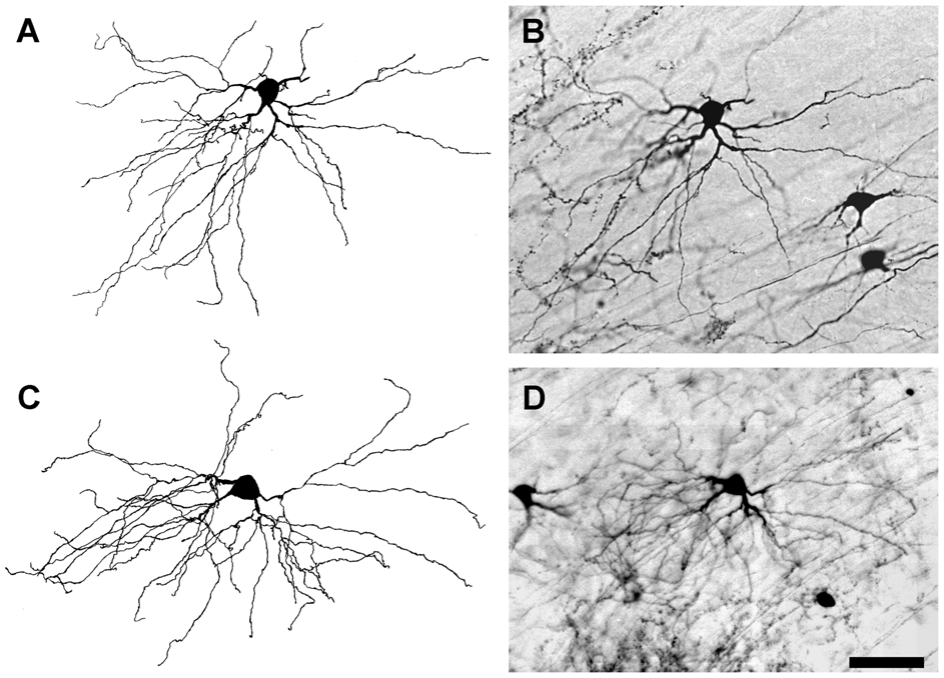
BDA-labeled basket cells. Basket cells are characterized by the asymmetrical distribution of their primary dendrites. (**A** and **C**) Camera lucida drawings of two representative basket cells. (**B** and **D**) Micrographs of the cells shown in **A** and in **C**, respectively. Scale bar: 50 µm.

Labeled projection neurons had 2–10 primary dendrites and up to 55 secondary and tertiary branches. Individual dendrites ranged in length from 30–300 µm. Primary dendrites usually had a smooth surface, a diameter of up to 7 µm, and tapered rapidly after emerging from the cell soma, giving rise to 2–6 secondary dendrites (Figures 3, 4, 5, and 6). Typically, dLGN projection neurons with large cell somas had more primary dendrites than those with small somas, but the correlation between cell soma size and dendritic number is modest (see below). Similarly large diameter primary dendrites extended more branches than small diameter dendrites. Secondary dendrites terminated without further branching or bifurcated into tertiary dendrites (arrowheads in Figure 3A). Typical secondary and tertiary dendrites were slender with a diameter of approximate 1 µm throughout their entire length. Axons were distinct from primary dendrites by their relatively small but even caliber (arrow in Figure 3C), and were often seen extending directly from cell somas. Occasionally, labeled axons could be traced for a short distance from the cell soma before leaving the plane of the section. On a few occasions, labeled axons were seen to branch after leaving the vicinity of the soma, and these collateral branches appeared to terminate within the dLGN.

#### Dendritic Appendages

Dendritic appendages were seen on all secondary or tertiary dendrites, and could be classified into three broad groups. More than 90% of the dendritic appendages observed were short in length and spaced irregularly along the distal segment of the dendrites (arrows in Figures 4A, B), with slightly higher density near the end of the dendrite. We refer to these appendages as tufted appendages. Seen less frequently were appendages that resembled spines. Spine-like appendages were seen on some but not all neurons. Each spine had a thin stalk and an ovoid head (arrows in Figures 4C, F), and was larger in overall size than typical tufted appendages. Spine-like appendages were usually distributed on the proximal or middle segments of secondary dendrites. In addition to tufted and spine-like appendages, appendages with grape-like clusters were occasionally observed emerging from the bifurcation of primary dendrites or along the proximal segments of secondary dendrites of neurons with medium or large cell somas. These appendages consisted of a short stalk and several ovoid terminal swellings (arrows in Figs. 4D, E).

### Morphological Classification of dLGN Projection Neurons

#### Classification

The dendritic arbors of many completely labeled dLGN projection neurons were not distributed uniformly around cell somas, and simple measurements of these arbors, e.g., diameter, do not define them accurately. Moreover, the dendritic arbors of some dLGN projection neurons did not conform precisely to typical geometric shapes, making it difficult to group these dLGN projection neurons according to the shapes of their dendritic arbors. Nevertheless, with these limitations clearly in mind, labeled dLGN projection neurons were classified morphologically into three broad classes: radial, bipolar, and basket cells. While most of the labeled projection neurons could be assigned readily to one of these three classes, the spatial distribution of the dendrites of approximately 3.3% of the labeled projection neurons that were classified did not conform unequivocally to a class-specific stereotype. For example, a few cells displayed contiguous primary dendrites emerging from approximately three-quarters of the cell soma circumference. Such a dendritic distribution does not match precisely the criteria used for classifying cells as radial cells or baskets cells. However, to avoid proposing additional classes of projections neurons that might include but one or two members in each class, drawings of the small number of projection neurons with nonconforming distributions of primary dendrites were scored by three independent observers, with the aid of additional information regarding the sometimes subtle structural differences among the three classes of projection neurons as presented in Table 1. The collective judgment of these three observers then was used to place the small number of nonconforming cells as a best fit into one of the three cardinal classes of projection neurons.

**Table 1:** Cytological Characteristics of the Three Classes of dLGN Projection Neurons.

		Radial Cells	Basket Cells	Bipolar Cells
Number of Primary Dendrites	Range	3–8	3–6	2–4
	Mean	4.88±0.12	4.36±0.14	2.42±0.19
Number of Dendrites	Range	12–55	10–44	16–34
	Mean	26.94±1.12	26.89±0.99	23.25±1.58
Cross-Sectional Area of Cell Soma (µm^2^)	Range	98–302	114–305	134–269
	Mean	200.0±5.64	205.7±7.66	180.3±13.29
Cross-Sectional Area of Dendritic Arbor (10^4^ µm^2^)	Range	1.248–6.958	1.279–5.897	1.723–4.487
	Mean	3.405±0.128	3.324 ± 0.123	2.938 ± 0.287

#### Radial Cells

**Figure 7:**
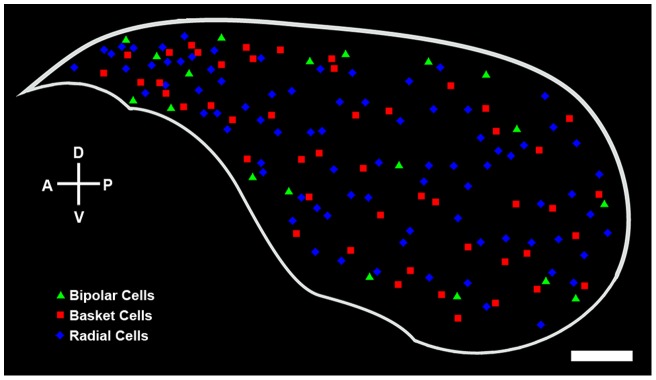
A three-dimensional reconstruction of the dLGN, which has been generated by stacking the coronal drawings of the dLGN in Figure S1 from anterior to posterior at 120 µm intervals and then mapping them in two dimensions, showing the spatial distributions of radial (blue diamonds), basket (red squares), and bipolar (green triangles) projection neurons. A: anterior, P: posterior, D: dorsal, V: ventral. Scale bar: 250 µm.

A cell was classified as radial if its dendrites were distributed around the cell soma without an apparent bias in any angular direction (Figure 3). Radial cells typically had at least 4 primary dendrites, evenly spaced relative to other dLGN cells, around the perimeter of the cell soma. The secondary and tertiary dendrites of radial cells were rich in tufted appendages, but only rarely were grape-like appendages observed. Radial cells comprised the largest population of labeled dLGN projection neurons (∼55%), and the analysis of the location of each BDA-labeled radial cell with respect to the borders of the dLGN in three dimensions indicated that radial cells are distributed evenly throughout the nucleus (Figures 7 and S1, Movie S1). However, radial cells varied significantly in cell soma size, total number of dendrites, and in the cross-sectional area of their dendritic arbors (Table 1).

#### Basket Cells

**Figure 8:**
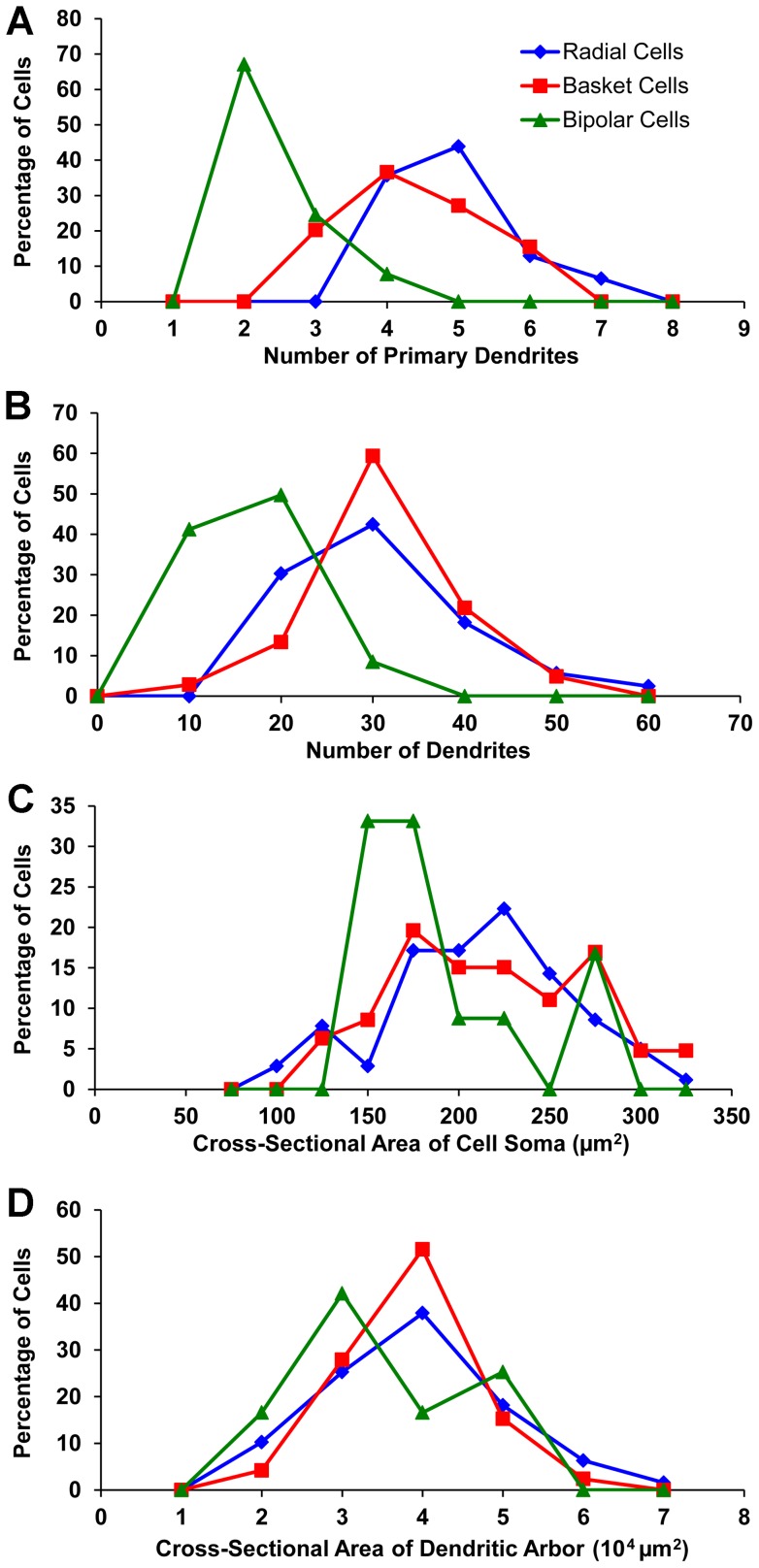
Cytological differences among the three classes of dLGN projection neurons. (**A**) Frequency distributions of the number of primary dendrites per cell, (**B**) the number of dendrites per cell, (**C**) the cross-sectional area of cell somas, and (**D**) the cross-sectional area of dendritic arbors.

Basket cells could be identified by their asymmetric distribution of dendrites predominantly from one side of the cell soma (Figure 6). The dendrites of basket cells were decorated primarily with tufted appendages, although spine- and grape-like appendages also were observed. Similar to radial cells, the three dimensional localization of labeled basket cells that was performed indicated that these cells also were distributed uniformly throughout the nucleus (Figures 7 and S1, Movie S1), and accounted for approximately 32% of the labeled dLGN projection neurons. Basket cells also varied significantly in the size of their cell somas and dendritic arbors and in the number of dendrites per cell (Table 1). Radial and basket cells were similar with respect to several parameters, namely the: (a) number of primary dendrites per cell; (b) total number of dendrites per cell; (c) mean cross-sectional cell soma area; and (d) mean cross-sectional of dendritic arbors (Figures 8A–D).

#### Bipolar Cells

Bipolar cells were characterized by having 2, or at most 4, primary dendrites that emerged from roughly opposite sides of the cell soma (Figure 5). The long-diameter of their dendritic arbors was aligned approximately parallel to the optic tract that courses over the dorsolateral surface of the dLGN. Grape-like appendages were found frequently on the proximal segments of dendrites. Tufted and spiny appendages also were observed on the relatively distal segments of dendrites. Bipolar cells typically had medium-sized somas and dendritic arbors (Table 1), and constituted about 13% of the labeled dLGN projection neurons. Bipolar cells were distributed preferentially near the dorsolateral or ventromedial surface of the dLGN although they also were seen in the center of the dLGN (Figures 7 and S1, Movie S1). Most bipolar cells displayed fewer primary dendrites and a smaller number of total dendrites than radial or basket cells (Figures 8A, B). In addition, the sizes of a majority of bipolar cell somas tended to be clustered in a narrow range, 150–175 µm^2^, whereas the cell soma sizes of radial and basket cells varied widely (Figure 8C).

### Distribution of dLGN Projection Neurons According to Cell Soma Size

**Figure 9:**
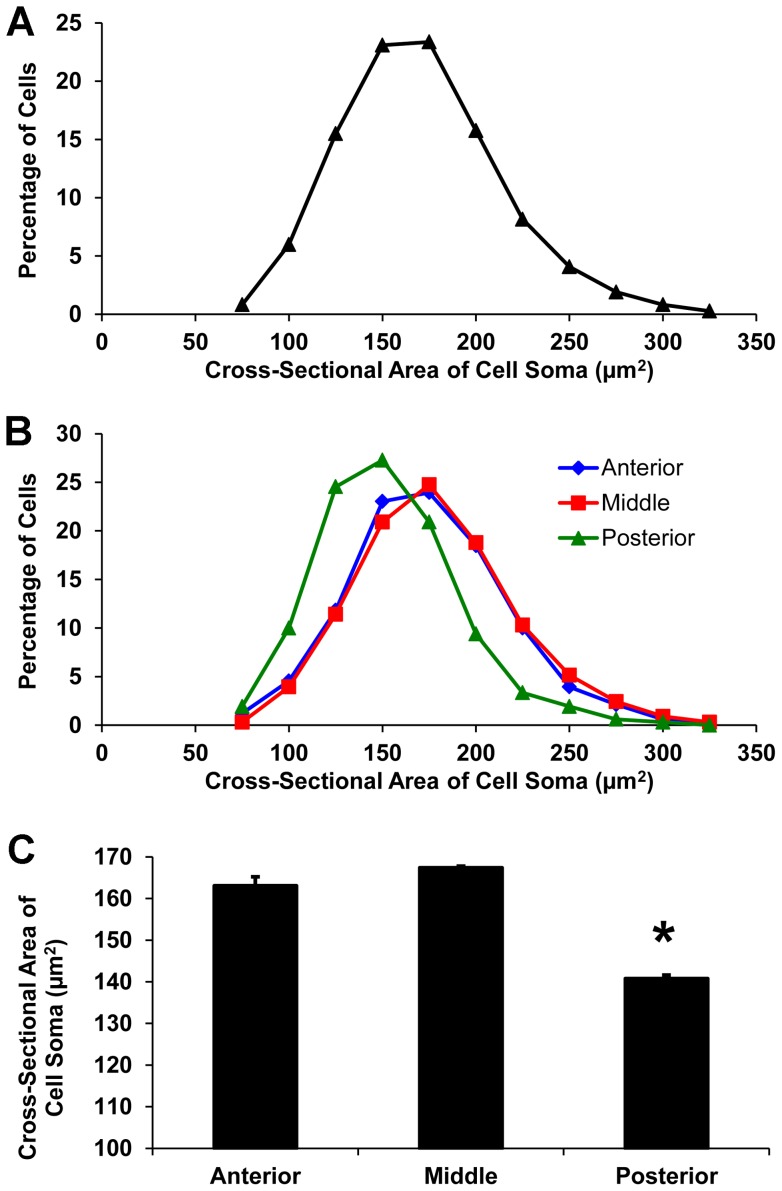
Cell soma size of dLGN projection neurons. (**A**) Frequency distribution of cell soma size. (**B**) Frequency distributions of cell soma size at three anterior-posterior planes of the dLGN: anterior (blue diamonds), middle (red squares), and posterior (green triangles). (**C**) Mean cell soma size at each of the three planes. The asterisk indicates that the mean cell soma size of projection neurons in the posterior plane of the dLGN is significantly smaller than that of cells in the middle or anterior level of the nucleus, p<0.01, two-tailed Student's *t*-test.

**Figure 10:**
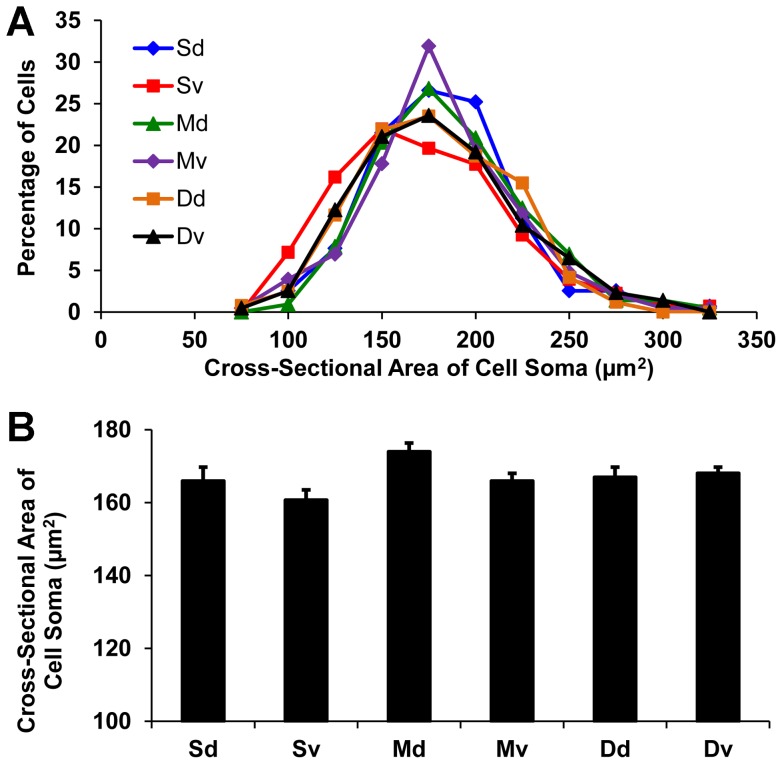
Cell soma size of dLGN projection neurons at six sectors near the rostro-caudal midpoint of the nucleus (**Figure 1B**). (**A**) Frequency distributions of cell soma size in each sector. (**B**) Mean cell soma size in each sector. No statistically significant differences were detected between any of the sectors that were analyzed.

The cross-sectional areas of the cell somas of over 4000 labeled dLGN projection neurons were measured at three A–P planes in the dLGN: anterior (A–P 2), middle (A–P 5), and posterior (A–P 8). Cell somas varied in size significantly, ranging from 60–340 µm^2^ (Figure 8C), although 99% of them were in the range of 75–300 µm^2^ (Figure 9A), with a mean cell soma size of 160 µm^2^. The distribution of cell soma size was similar in both the anterior and the middle planes of the nucleus (Figure 9B). However, the distribution of cell soma size in the posterior plane was shifted significantly toward smaller sizes. Within the anterior and middle planes of the dLGN, over 60% of the projection neurons had cell somas with cross-sectional areas that ranged from 150 µm^2^ to 200 µm^2^ (Figure 9B). By contrast, a majority of the projection neurons in the posterior plane had smaller cell somas with cross-sectional areas of 125 µm^2^ to 175 µm^2^ (Figure 9B). Thus, labeled projection neuron cell somas in the anterior and middle thirds of the dLGN had cross-sectional areas of comparable size, but projection neurons located in the posterior third of the dLGN had significantly smaller cell somas (Figure 9C; p<0.01, two tailed *t*-test). The distribution of dLGN neurons according to cell soma size in the six sectors of the dLGN at A–P plane 5 (Figure 1B) also was investigated. Both the distribution (Figure 10A) and the mean cross-sectional areas of cell somas (Figure 10B) were similar among the sectors.

### Projection Neuron Cell Soma Size, Dendritic Arbor Size, and the Number of Dendrites per Cell

**Figure 11:**
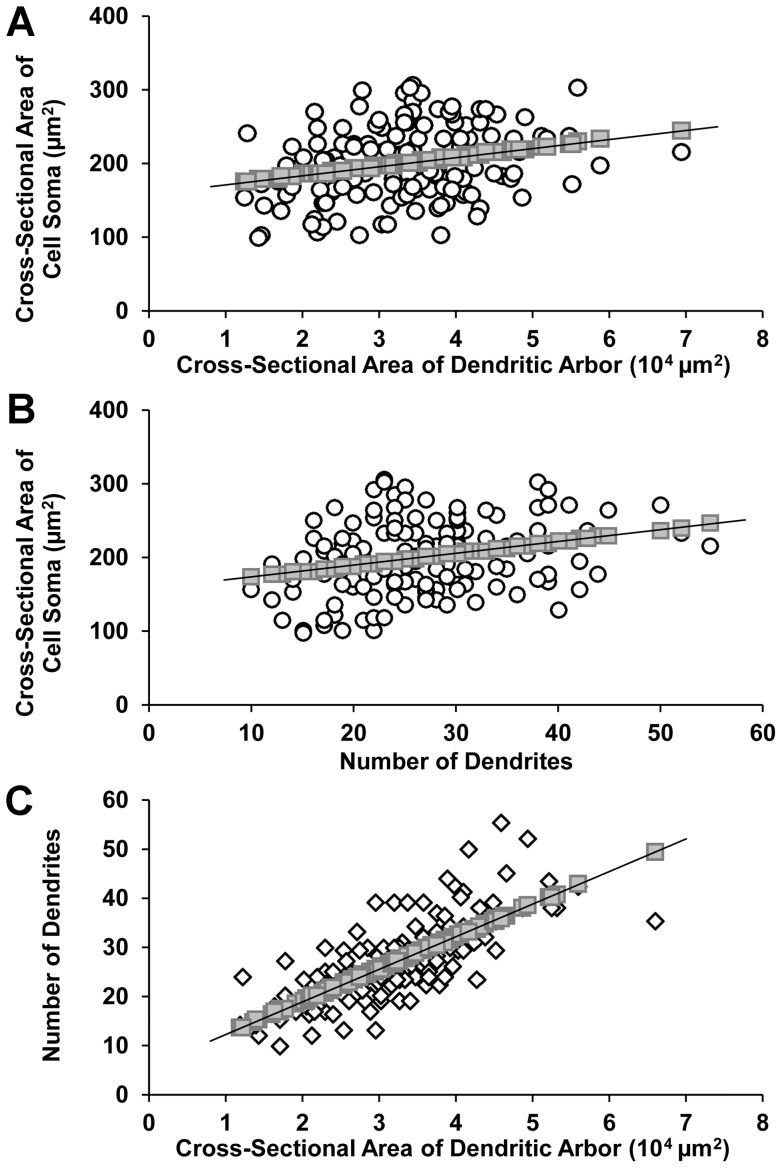
Correlation between cell soma size, number of dendrites per cell, and cross-sectional area of dendritic arbors of dLGN projection neurons. There are relatively weak positive correlations between (**A**) cell soma size and dendritic arbor size (r = 0.25*) and (**B**) between cell soma size and the number of dendrites per cell (r = 0.28*). (**C**) In contrast, a relatively strong positive correlation exists between the number of dendrites per cell and the size of the cross-sectional area of the dendritic arbor (r = 0.75**). * p<0.01, ** p<0.001, two-tailed Student's *t*-test.

The number of primary dendrites and total number of dendrites for each class of dLGN projection neuron revealed a clear modal value for neurons in each class (Figures 8A, B). Cross-sectional areas of projection neuron dendritic arbors varied from 1.24×10^4^ µm^2^ to 6.96×10^4^ µm^2^ (Figure 8D). However, differences among cell classes with respect to the number of dendrites, and the cross-sectional area of cell somas or dendritic arbors were not statistically significant. By contrast the number of primary dendrites differed significantly between radial and basket cells (p<0.01, two-tailed *t*-test), and between radial or basket cells and bipolar cells, p<0.001, two tailed *t*-test (Table 1). When the sizes of dendritic arbors, or the number of dendrites, were plotted against the sizes of cell somas (Figures 11A, B), a positive, but weak correlation between each pair of variables was observed, p<0.01, two-tailed *t*-test, indicating that the cross-sectional area of the cell soma of an dLGN projection neuron is not a strong predictor of the dendritic number or arbor size. By contrast, there was a strong positive correlation between the size of dendritic arbors and dendrite number (Figure 11C; r = 0.75, p<0.001, two-tailed *t*-test), indicating that large dendritic arbors generally comprise more dendrites than small ones. The frequency distributions related to dendritic number, arbor size and cell soma size have not been analyzed statistically, and are included only for the purpose of illustrating a trend where one exists, e.g., Figure 8A, or does not, e.g., Figure 10A.

## Discussion

In this report we have described the complete morphology of 149 identified projection neurons in the dLGN of the adult rat, and examined the size and distribution of over 4000 projection neuron cell somas in the dLGN. Leaving aside a single report that concluded that dLGN neurons in the rat cannot be classified according to their structure [9], many studies have proposed classification schemes predicated upon observed differences in the morphology of rat dLGN neurons. Golgi studies in the adult and developing rat, have grouped dLGN into two classes, A and B, based upon cell soma size and dendritic pattern, and have assumed that Class A cells are projection neurons and Class B cells are interneurons [3], [7]. Kriebel [8] also used Golgi preparations of the rat dLGN and concluded that three types of neurons could be distinguished given differences in cell soma size, dendritic structure and a cell's location in the dLGN. Kriebel speculated that the cells classified as type 1 or type 2 neurons might be projection neurons. However, the fundamental distinction between projection neurons and interneurons is unrelated to cell soma size or dendritic pattern, but instead depends upon whether a neuron's axon terminates locally in the vicinity of the cell soma or at a distance from it.

Unfortunately, this distinction cannot be made unequivocally in Golgi preparations, and, therefore, the use of horseradish peroxidase (HRP) as a retrograde tracer to label dLGN projection neurons in the rat provided the first description of a confirmed projection neuron in the rat dLGN [10]. While a step forward, this report illustrates only one type of HRP-labeled projection neuron, which appears comparable to the radial cells that we have described here. It is possible, however, that only one class of projection neuron was described because the retrogradely transported HRP may not have filled all cells solidly or some of the filled cells may have been obscured by artefacts as illustrated in Figures 5A and 5B of [10]. In almost all of these previous reports, it is implied that most if not all dLGN projection neurons have large cell somas and dendritic arbors in contrast to presumptive interneurons that are thought to have relatively small cell somas and few dendrites. The present results indicate that these assumptions should be viewed with caution as we have observed dLGN projection neurons with small cell somas and few dendrites, or with medium-sized cell somas that are similar in size to dLGN interneurons that have been identified electrophysiologically [18].

Our results show that the morphology of dLGN projection neurons in the rat is diverse, and, therefore, cell soma size and dendrite number are not reliable criteria per se for discriminating between projection neurons and interneurons. In addition, the present results demonstrate that the dendrites of some projection neurons in the rat dLGN may extend up to 300 µm from the cell soma, resulting in a dendritic arbor with a cross-sectional area of up to 7.0×10^4^ µm^2^, 30% larger than previously reported in the rat and much greater than that which has been reported for presumed projection neurons in the mouse dLGN [40]. Such large dendritic fields suggest that some dLGN projection neurons in the rat may receive input from much wider areas of the retina than previously considered, and that the coverage of the visual field by these large dendritic arbors may overlap significantly with the coverage of neighboring dLGN projection neurons. Although neighboring projection neurons with large overlapping dendritic fields would be detrimental to resolution, they would ensure a high degree of redundancy in covering visual space and would enhance visual function at low light levels.

We propose that three classes of projection neurons can be found in the rat dLGN, based on clear differences in the spatial distribution of the dendrites of projection neurons. Despite variability within each cell class, the members of each of the three classes share a common affinity in the spatial organization of their dendritic arbors. Radial cells have dendrites that are arrayed around the entire perimeter of the cell soma; bipolar cells have dendrites that typically arise from just two locations on the cell soma that lie opposite each other; and basket cells have dendrites that emerge largely along one side of the cell soma. Three classes of dendritic appendages also are described: tufted, spiny and grape-like. All sizes and classes of the dLGN projection neurons appear to support tufted appendages, but spiny appendages are found only on the dendrites of medium and large cells. Grape-like appendages are seen frequently on bipolar cells, occasionally on basket cells, and rarely on radial cells.

The results presented here indicate that the distribution of the dendrites around the cell soma in typical Class A or type 1 projection neurons [3], [7], [8] is comparable to that of the projection neurons that we have classified as radial cells. However, radial cells are not restricted to neurons with large cell somas and can include small and medium dLGN relay cells. Type 2 neurons resemble cells that are classified here as basket cells. However, basket cells are found throughout the anterior-posterior extent of the dLGN, and are not localized superficially in the middle third of the dLGN, as reported for type 2 cells [8]. Thus, the earlier classification of presumptive dLGN projection neurons and the classification described here differ. Moreover, the present results demonstrate that a third class of projection neuron, which we have termed bipolar cells, can be identified in the rat dLGN and displays a characteristic asymmetrical distribution of dendrites around the cell soma that is not shared by cells classified as radial or basket. Bipolar projection neurons resemble Class B cells which have been presumed to be interneurons [3], [7], [9], and while interneurons generally may be bipolar in configuration, this cytological organization is not unique to interneurons in the rat dLGN. Thus, the most salient difference between earlier classification schemes of dLGN neurons in the rat dLGN and the one presented in this report is that the current results define three distinct classes of dLGN projection neurons in contrast to one [3], [7] or two [8] classes.

Projection neurons in the dLGN of the rat also have been grouped according to their retinal input and physiological response properties [12]–[18], [26]. Large cells receive afferents mainly from retinal type 2a axons, while small and medium cells receive afferents from type 2b retinal axons [26]. Since dLGN projection neurons are activated monosynaptically by retinal afferents, these neurons also have been classified as fast or slow cells, respectively, based on the latency of their response to electrical stimulation of the optic chiasm. Fast cells receive their input from retinal ganglion cells with fast conducting axons while slow cells receive input from ganglion cells with slowly conducting axons [14], [15]. Moreover, fast cells predominate in the rostroventromedial region of the dLGN, and slow cells in the caudal part of the nucleus (17).

In this study, we analyzed the distribution of dLGN projection neurons in relation to cell soma size. Although projection neurons with cell somas of different sizes are intermingled throughout the dLGN, projection neurons in the caudal dLGN, which has been reported to comprise more slow cells than fast ones, were significantly smaller in size than neurons located in the middle or rostral regions of the dLGN. However, the mid-ventral, Mv, sector of the dLGN (Figure 1B), a region thought to contain a majority of fast cells [17], did not differ with respect to cell size distribution from any of the other sectors in the dLGN. Similarly, while fast and slow cells are found in rostrodorsolateral region of the dLGN, slow cells represent two thirds of the population of projection neurons in this region [17]. However, the cell soma sizes of projection neurons in the rostral region of the dLGN are not skewed toward small and medium sizes. Taken together, these data suggest that there may be a correlation between dLGN relay cell soma size and response latency to optic tract stimulation, but it does not appear to be strong.

As mentioned, classifying dLGN projection neurons in the rat by cell soma size is not reliable, as neurons in distinctly different structural classes, as described here, may have similar cell soma sizes. By contrast, the classification of dLGN projection neurons in this study is based solely on a detailed analysis of the dendritic structure of individual projection neurons. That such a classification is not always consistent with the functional specificity of neurons is to be expected, as such specificity can be influenced by factors that are independent of a neuron's intrinsic structure, such as the conduction velocity of afferents to the neuron.

Noteworthy is a recent report in the mouse [40] that describes three classes of presumed projection neurons in the dLGN. The cells described in this paper had been reconstructed after being injected with biocytin in 250–300 µm thick slices through the dLGN, and they appear largely identical with respect to the general organization of their dendritic arbors in the three classes of confirmed dLGN projection neurons in the rat that are illustrated here. Of particular interest is the greater breadth of the dendritic arbors of the confirmed dLGN projection neurons in the rat that are described in this report, with the breadth of the dendritic arbors of dLGN neurons in the mouse that appear to be comparable [40].

In Golgi preparations of the cat dLGN, Guillery [41] described three classes of dLGN cells in the A laminae of the nucleus. Class 1 and Class 2 cells closely resemble radial and basket cells respectively. Some Class 3 cells, which were seen infrequently, bear a passing resemblance to bipolar cells, but Class 3 cells appear to be more varied with respect to the disposition of their dendrites than bipolar cells. Combining electrophysiology with the intracellular injection of HRP, Friedlander et al. [42] determined the structure and function of 47 neurons in the cat dLGN. Of the cells illustrated, radial- and bipolar-like cells, often with more elaborate dendritic arbors than those in the rat dLGN, appear to have been frequently observed, but few basket-like cells were seen, perhaps because of the sample size.

While it is reasonably clear that presumed projection neurons in the mouse, and confirmed projection neurons in the rat, and cat can be grouped broadly into three similarly distinct classes of cells based on dendritic arbor structure, the morphology of neurons in the dLGN of the monkey and human have been studied only with Golgi impregnation and, therefore, it has not been possible to distinguish projection neurons from interneurons. Nevertheless, drawings of Golgi-stained neurons the monkey [43], [44] and human [45], [46] dLGN illustrate cells that resemble the radial, basket and bipolar cells described here, and one might cautiously speculate that these cells are dLGN projection neurons. However, confirmation awaits a study that fully labels identified geniculo-cortical projection neurons in the monkey and provides an empirical basis for extrapolation to humans.

In summary, the classification system presented here takes into account structural differences in the dendritic arbors of dLGN projection neurons in the normal adult rat that allows these neurons to be sorted into three classes. This classification scheme provides a reliable morphological description of identified dLGN projection neurons in normal adult rats. Such a description is essential in evaluating the changes that may occur in dLGN projection neurons after injury, and in determining whether projection neurons respond uniformly or differentially according to class in response to a common insult such as axotomy.

## Supporting Information

Figure S1 **Montage of coronal sections through the dLGN showing the locations of confirmed radial (blue diamonds), basket (red squares), and bipolar (green triangles) projection neurons.** The A–P position of each section relative to bregma is indicated below each section. The lower right panel displays an overlay of all sections in the series. D: dorsal, V: ventral, M: medial, L: lateral. Scale bar: 250 µm.
(TIF)

Movie S1 **Three-dimensional rotating image about the D-V axis of the coronal sections and projection neurons illustrated in Figure S1.** The spacing between adjacent sections is 120 µm.
(AVI)
