# Supplementary figures and images for: Morphology, Classification, and Distribution of the Projection Neurons in the Dorsal Lateral Geniculate Nucleus of the Rat
Source: PLoS One. 2012 Nov 5;7(11):e49161. doi: 10.1371/journal.pone.0049161 (PMC3489731; doi:10.1371/journal.pone.0049161)

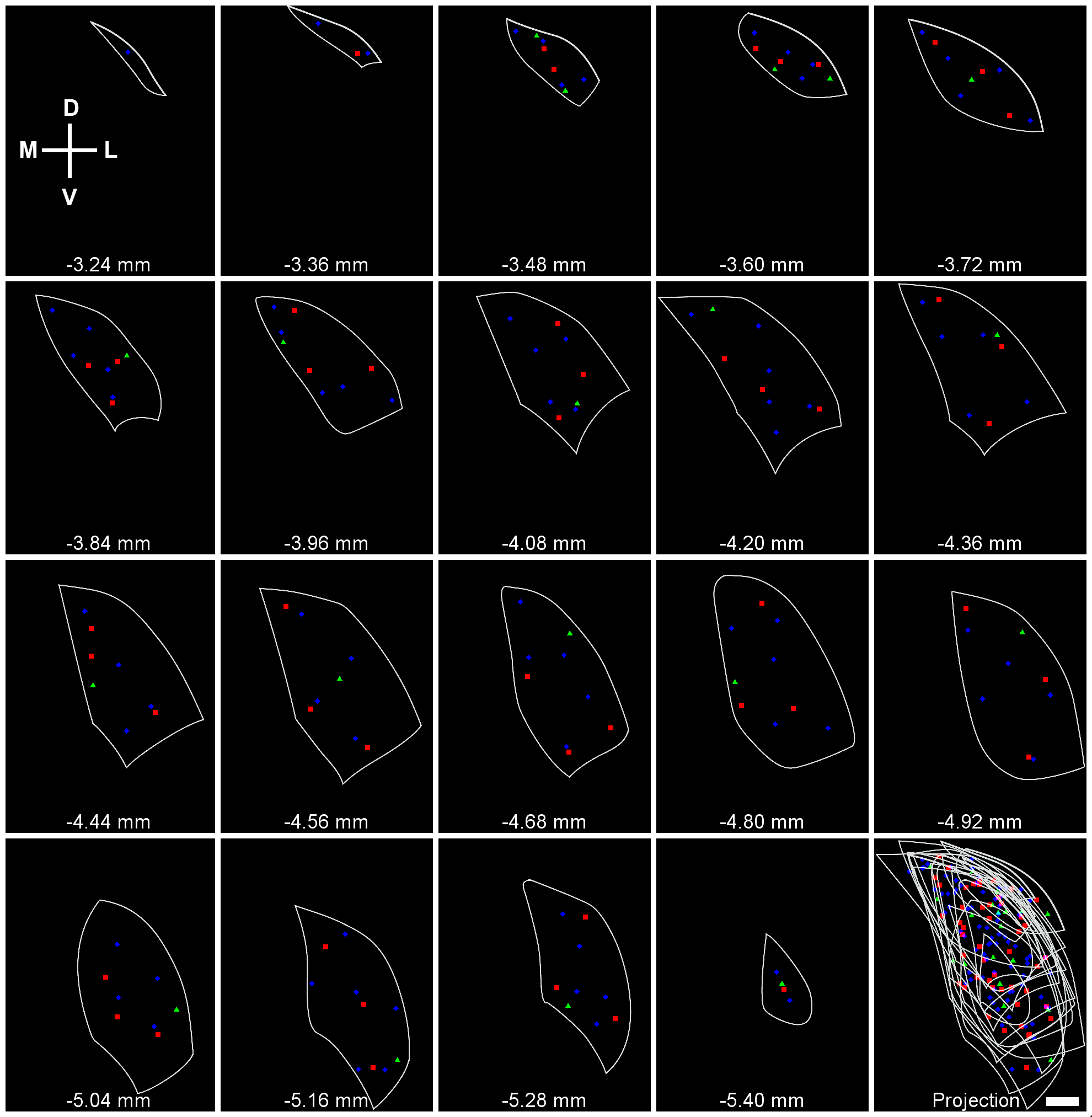

Supplement: Figure S1 — Montage of coronal sections through the dLGN showing the locations of confirmed radial (blue diamonds), basket (red squares), and bipolar (green triangles) projection neurons. The A–P position of each section relative to bregma is indicated below each section. The lower right panel displays an overlay of all sections in the series. D: dorsal, V: ventral, M: medial, L: lateral. Scale bar: 250 µm. (TIF) [file pone.0049161.s001.tif]
